# An unusual presentation of sixth nerve palsy: neurobrucellosis

**DOI:** 10.3205/oc000102

**Published:** 2019-04-04

**Authors:** Burak Mergen, Ahmet M. Sarici, Fatih Baltu, Resat Ozaras, Ibrahim Adaletli

**Affiliations:** 1 Deparment of Ophthalmology, Cerrahpasa Medical Faculty, Istanbul University, Istanbul, Turkey; 2 Department of Infectious Diseases and Clinical Microbiology, Cerrahpasa Medical Faculty, Istanbul University, Istanbul, Turkey; 3 Department of Radiology, Cerrahpasa Medical Faculty, Istanbul University, Istanbul, Turkey

**Keywords:** neurobrucellosis, abducens nerve palsy, meningitis

## Abstract

Abducens nerve palsy is generally seen in older patients with diabetes and hypertension. It is relatively rare in young and otherwise healthy patients. An extensive differential diagnosis is considered in patients younger than 50 years of age who develop a sixth nerve palsy. We report here a 25-year-old patient from Turkey who was admitted with sixth nerve palsy as a component of neurobrucellosis. She was referred to our clinic because she had double vision and restricted right eye abduction. During the ophthalmic examination, both pupils were round and reactive to light and accommodation. Extraocular movements were intact with the exception that the right eye was unable to abduct. Magnetic resonance imaging revealed no pathology. She also had a diagnosis of brucellosis and her abducens nerve palsy was a form of clinical manifestation of neurobrucellosis. We conclude that neurobrucellosis should be considered in patients with sixth cranial nerve palsy especially in areas where brucellosis is endemic.

## Introduction

Abducens nerve palsy is generally seen in old patients with diabetes and hypertension [1]. In the largest case series of cranial nerve palsies done through the Mayo clinic, the cause was most frequently undetermined; neoplasm, head trauma and vascular causes were next in frequency [2]. It was also shown that patients in whom the etiology was known were younger than the patients with undetermined cause.

Brucellosis is a zoonosis still endemic around Mediterranean countries, the Middle East, Mexico, and Central and South America. Involvement of various cranial nerves may be seen very rarely as a component of neurobrucellosis. There are few cases of abducens nerve palsy which were reported in previous reports [3], [4], [5].

Here we report a neurobrucellosis patient with an unusual presentation of sixth nerve palsy.

## Case description

**Figure 1:**
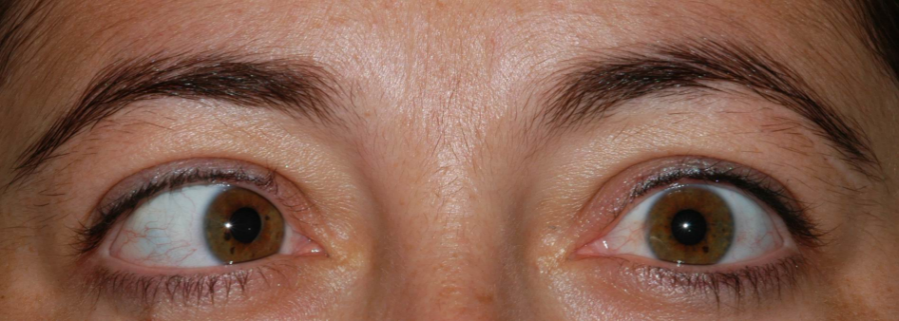
The patient had right esotropia in primary position.

**Figure 2:**
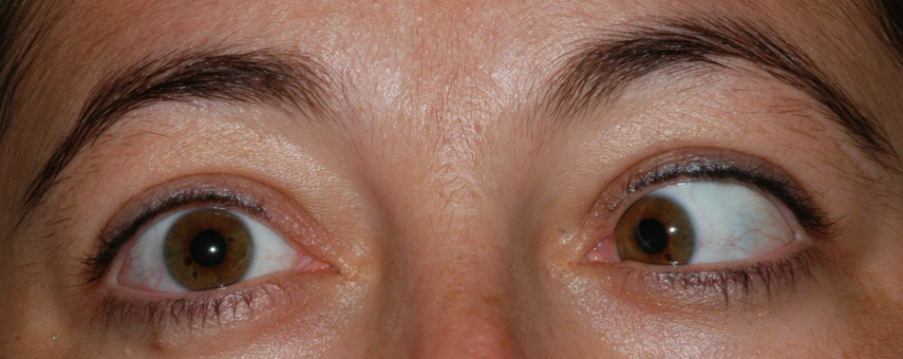
The patient had a restricted right gaze that revealed a right abducens palsy.

A 25-year-old female was referred to our clinic with double vision and restricted right eye abduction. In the ophthalmic examination, both pupils were round and reactive to light and accommodation. Extraocular movements were intact with the exception that the right eye was unable to abduct (Figure 1 (Fig. 1), Figure 2 (Fig. 2)). No nystagmus was detected and visual acuity was normal. Visual field examination revealed the enlargement of the blind spot and constriction of the peripheral fields. On fundus examination, bilateral papilledema with blurring of the disc margins was present.

Questioning her history revealed that the first symptoms were weakness and numbness in her lower limbs which had resolved spontaneously. Afterwards, she had developed an intractable headache associated with right gaze difficulty and diplopia and was referred from the emergency room to the neurology department. Magnetic resonance (MR) imaging revealed no pathology. After our finding of papilledema, despite the history of transient weakness in the lower limbs, the cause was firstly considered as idiopathic intracranial hypertension (IIH) and lumbar puncture was planned. But because she developed fever and neck rigidity at that time, she was referred to the department of infectious diseases to rule out meningitis.

The patient underwent a lumbar puncture, opening pressure was 255 mm H_2_O and the examination of the cerebrospinal fluid (CSF) showed 85 leukocytes/μl. Her Rose Bengal test was positive and the CSF culture yielded *Brucella melitensis* and diagnosis of neurobrucellosis was established. The patient was treated with a combination of doxycycline, rifampin, cotrimoxazole, and dexamethasone for 6 months.

## Discussion

The etiology of most of the sixth cranial nerve palsies remains unclear and the patients in whom the cause is revealed tend to be younger. The most frequent reasons after the idiopathic cases were found to be neoplasms, head trauma, and vascular causes [2].

Our patient had sixth nerve palsy plus the history of weakness in the lower limbs. MRI findings showed us no pathology, so the diagnosis of IIH was considered and lumbar puncture was planned. But when neck rigidity occurred, she was referred to the department of infectious diseases, underwent a lumbar puncture test and the CSF culture was positive for brucellosis. These findings suggest that abducens palsy was due to neurobrucellosis.

Brucellosis is still a major infectious disease issue in many regions of the world, especially in Mediterranean countries, the Middle East and South America. Transmission of the disease to humans occurs by contact with meat or placenta of infected animals or ingestion of unpasteurised milk or cheese. Brucellosis is a multisystem disease with various clinical presentations. Neurobrucellosis is an important clinical manifestation of systemic brucellosis and was reported in 3–13% of patients with brucellosis [6]. The most frequent clinical presentations of neurobrucellosis are meningitis, meningoencephalitis, myelitis, polyradiculitis, and meningovascular syndromes [7]. Involvement of various cranial nerves may accompany neurobrucellosis. A few cases have been reported with abducens nerve palsy as a manifestation of neurobrucellosis [5], [8].

Mechanism of sixth nerve palsy during neurobrucellosis could be due to meningitis. However, although any meningitis can cause sixth nerve palsy either through direct inflammation, increased ICP, or both, our patient had developed sixth nerve palsy before the symptoms of meningitis developed. Thus the involvement of the cranial nerve might have started before the involvement of meninx. Our case presented with sixth cranial nerve palsy before the diagnosis of neurobrucellosis, however Yilmaz et al. reported a case in whom palsy was developed during the treatment of neurobrucellosis [5]. Sahin et al. also showed that multiple cranial nerves including optic, vestibulocochclear, and abducens can also be involved in neurobrucellosis [9]. Although the opening pressure was high, the papilledema of our patient might also be related with direct involvement, but bilaterality decreases this possibility. Unilateral optic neuritis and abducens nerve involvement as initial findings in neurobrucellosis were also reported [10].

Patients with acute sixth nerve palsy must undergo a complete neurologic and medical evaluation [2]. Myasthenia, para-infectious disease, trauma, and tumors must be considered firstly and syphilis, sarcoidosis, or Lyme’s disease might be considered as rare causes. Neuroimaging and CSF analysis should be suggested when other neurologic symptoms associate. Although the idiopathic group was found to be observed as the most frequent reason [2], there might be various reasons that we miss revealing the cause of the sixth nerve palsy. Brucellosis was not taken into consideration previously when investigating sixth nerve palsy but neurobrucellosis might be a cause for the cases that were classified as undetermined or idiopathic.

In conclusion, we suggest that evaluation of sixth nerve palsy should include tests for brucellosis as well, especially for patients who live in endemic areas. Neurobrucellosis should also be kept in mind when considering patients with intractable headaches and cranial nerve palsies.

## Notes

### Competing interests

The authors declare that they have no competing interests.
